# Osseointegration of Plasma-Modified Titanium Dental Implants Enhanced by Maca Nano-Suspension in New Zealand Rabbits: An Animal Study

**DOI:** 10.1007/s00784-026-07210-1

**Published:** 2026-11-10

**Authors:** Hesham Fathi Manie, Heba Ahmed Saleh, Mai El Halawany, Rania Salama, Mohamed Mokhtar Khashaba

**Affiliations:** 1 Department of Oral and Maxillofacial Surgery, Faculty of Dentistry, Cairo University, Cairo, Egypt; 2 Department of Oral and Maxillofacial Pathology, Faculty of Dentistry, Cairo University, Cairo, Egypt; 3 Department of Pharmaceutics and Industrial Pharmacy, Faculty of Pharmacy, Cairo University, Cairo, Egypt; 4 Department of Biomaterials, Faculty of Dentistry, Cairo University, Cairo, Egypt

**Keywords:** Implant stability, Maca, Cold atmospheric plasma, Bone regeneration, Histomorphometric analysis, Animal study

## Abstract

**Objectives:**

To investigate the potential effect of combined cold atmospheric plasma and Maca extract nano-suspension as a dual implant surface treatment on osseointegration.

**Materials and methods:**

Maca nano-suspension was formulated and characterized. Thirty titanium implants were placed in the tibiae of ten adult male New Zealand rabbits and were divided into three groups: untreated, cold atmospheric plasma-treated implants, and cold atmospheric plasma + Maca extract-treated implants. Implant stability was assessed using the AnyCheck^®^ device at the time of placement (primary stability), and then after four weeks (secondary stability). Histomorphometric analysis of the processed bone specimens was performed. Statistical significance level was set at *P* ≤ 0.05.

**Results:**

No statistical significance difference was noted regarding the primary stability between the groups, whereas the secondary stability was significantly greater in the cold atmospheric plasma-treated group compared with the untreated control group (*P-*value = 0.015). Histomorphometric analysis showed significantly greater percentages of mineralized bone area around implants in the cold atmospheric plasma + Maca group compared with other groups.

**Conclusion:**

The combination of cold atmospheric plasma and Maca extract resulted in a greater area percentage of newly formed mineralized bone, suggesting a potential beneficial effect on peri-implant bone regeneration.

## Introduction

Dental implants are an effective treatment option for replacing missing teeth, with high rates of long-term survival and success. However, achieving and maintaining reliable osseointegration remains a critical determinant of implant stability and overall treatment outcome. Osseointegration is defined as the direct structural and functional connection between living bone and the surface of a load-bearing implant, and it depends on both biological and mechanical factors. Multiple clinical trials suggested that early implant failure is most often associated with insufficient or delayed osseointegration, highlighting the importance of optimizing the implant–bone interface [1].

Primary implant stability is achieved mechanically at the time of placement, but it gradually decreases as bone remodeling begins. Meanwhile, the secondary stability, which develops through new bone formation and altered bone around the implant surface, is reported to be essential for the long-term success of clinical cases [2]. Several surface modification techniques have been introduced to accelerate and enhance this process, including sandblasting, acid etching, anodization, and the application of bioactive coatings [3]. These approaches have shown a positive impact on osseointegration and, consequently, on early stability processes. Yet, limitations such as increased cost, complexity, and potential material degradation remain challenging for widespread application of such techniques [2, 4].

Surface treatments such as cold atmospheric plasma (CAP) have gained worldwide attention in implant dentistry. CAP is a non-thermal ionized gas that can effectively modify the implant surface by removing organic contaminants, enhancing wettability, and increasing surface energy. These modifications promote protein adsorption, osteoblast adhesion, and subsequent bone formation [5]. Currently, CAP treatment has the advantage of being applied chair-side, immediately before implant placement, avoiding the undesirable hydrophobic recovery, which has been reported as a limitation in earlier plasma-based techniques [6]. Several clinical and preclinical studies have reported enhanced secondary stability and bone-to-implant contact following plasma activation [7–9].

Nowadays, there is a worldwide growing interest in the use of natural bioactive agents to support bone regeneration as Ashtiani et al. (2022) demonstrated improved bone-to-implant contact using different herbal extract coatings on titanium surfaces, such as grape seed extract [10]. Lepidium meyenii (Maca root) has attracted attention nowadays due to its purported osteogenic, antioxidant, and anti-inflammatory properties. Pre-clinical studies on Maca have identified constituents that mainly stimulate osteoblast proliferation, differentiation, and mineralization, and up-regulate osteogenesis-related genes such as BMP-2, ALP, and CBFα1 [11]. A comprehensive study by Beharry & Heinrich (2018) reported that Maca has shown positive effects on bone metabolism, including increased bone mineral density, femur weight, blood gla protein levels, and reversal of ovariectomy-induced trabecular bone loss in a rat model [12]. Furthermore, Carpio et al. (2024) concluded that Maca has potent anti-inflammatory properties by reducing inflammatory mediators, modulating cytokine responses, through its immunoregulatory effects [13]. More recently, Abdoun et al. (2026) demonstrated the osteogenic and anti-inflammatory effects of Maca in a rabbit calvarial bone defect model, reporting enhanced formation of newly formed mineralized and unmineralized bone tissue [14]. Collectively, these findings provide further support for the potential role of Maca in promoting bone regeneration. However, despite the growing evidence regarding its effects on bone metabolism and regeneration, its potential application as an adjunct to dental implant surface modification in vivo remains unexplored. Therefore, the current study aimed to investigate the effect of combining cold atmospheric plasma with a nano-formulated Maca root extract as a novel combination, which may have a positive impact on the osseointegration of titanium dental implants, as an initial in-vivo investigation.

## Materials and methods

### Study settings and ethical considerations

The current animal study was conducted in the animal house, Faculty of Medicine, Cairo University. All animal procedures were approved by the Institutional Animal Care and Use Committee (IACUC), Cairo University (Approval No. CUIIIF39a24), and were performed in accordance with institutional guidelines and the ARRIVE guidelines.

### Sample size calculation and study groups

Given the limited published evidence directly applicable to the present combination of CAP and Maca nanosuspension, the study was designed as a pilot in-vivo investigation to provide preliminary data on the potential effects of the proposed intervention. A total of thirty titanium implants (Dual Implant, Titan Industries, Egypt) were surgically inserted into the tibiae of 10 New Zealand rabbits bilaterally. The implants were allocated into three experimental groups (*n* = 10 implants per group): untreated implants as a negative control group (CN), CAP-treated implants as a positive group (CP), and implants with combined CAP and Maca extract treatment as an intervention group (CP-M).

### Eligibility criteria

In accordance with the ARRIVE guidelines, this experimental study was conducted on ten adult male New Zealand rabbits. Adult rabbits meeting the predefined eligibility criteria (age > 6 months and body weight 2.5–3.0 kg) were enrolled in the study, with mean age and body weight of 7.9 ± 0.9 months and 2.78 ± 0.15 kg, respectively. Rabbits were excluded if they were female, immature, or exhibited any signs of systemic or local disease.

### Maca extract preparation

#### Extraction procedure

An initial extraction was performed by combining approximately 750 mg of Maca powder with 200 mL of 70% ethanol [15]. This mixture was placed in a bath sonicator (275 T, Crest Ultrasonic Corp., Trenton, USA) for 4 h and then refrigerated for 24 h to allow the suspension to settle. The liquid extract was decanted, and the remaining solid residue was then mixed with an additional 100 mL of 70% ethanol to repeat the extraction steps and collect a second batch [16, 17]. After the second decantation, the leftover solid was discarded. The combined extracts underwent evaporation using a rotary evaporator (Heidolph, Heizbad Hei-VaP^®^, Heidolph, instruments GmbH & Co., Schwabach, Germany) set at 60 °C and 90 rpm for 1 h under vacuum to remove ethanol, resulting in a thin film [18]. This film was then rehydrated with 20 mL of distilled water, stirred at atmospheric pressure at 150 rpm for 30 min. Finally, the rehydrated extract was frozen overnight at − 21 °C, followed by freeze-drying for 24 h at − 60 °C and 0.1 atm to yield the final lyophilized Maca powder [19].

#### Preparation of Maca nano-suspension (Maca-NS)

The Maca nano-suspension (Maca-NS) was formulated using a liquid anti-solvent precipitation approach adopted by Gera et al. (2017) [20]. Approximately 40 mg of lyophilized maca extract was dissolved in 2 mL of methanol to constitute the (organic phase), while the aqueous anti-solvent (inorganic phase) was prepared by dissolving 200 mg hydroxypropyl methylcellulose (HPMC) and 200 mg Pluronic^®^ F-127 in 20 mL of distilled water. UndSper magnetic stirring (WiseStir MSH-30D, Wise laboratory instruments, DAIHAN Scientific Co., USA) at 1,000 rpm, the organic solution was introduced dropwise into the aqueous phase to induce supersaturation and nucleation. The resulting dispersion was sonicated for 15 min to enhance nanoparticle formation and prevent aggregation, as recommended in protocols involving solvent anti-solvent systems. Subsequently, the mixture was left stirring for 2 h at ambient conditions to facilitate the complete evaporation of methanol. The nano-suspension was then refrigerated until further use [20].

#### Characterization of the Maca-NS

The mean particle size (PS), polydispersity index (PDI), and zeta potential (ZP) of the prepared Maca-NS were determined via Dynamic Light Scattering (DLS) by means of a Zetasizer^®^ Nano ZS (Malvern Instruments, Worcestershire, UK). In order to achieve optimal signal intensity, 0.1 mL of the formulation was diluted to 10 mL with deionized water. Each test was performed in triplicate [21, 22].

### Housing and husbandry

Rabbits were housed under a controlled environment (temperature 25 ± 2 °C and 12-hour dark/light cycles). They were kept individually in clean stainless-steel cages, with a standard rabbit feeding diet and fresh tap water [23].

### Surgical procedure

**Fig. 1:**
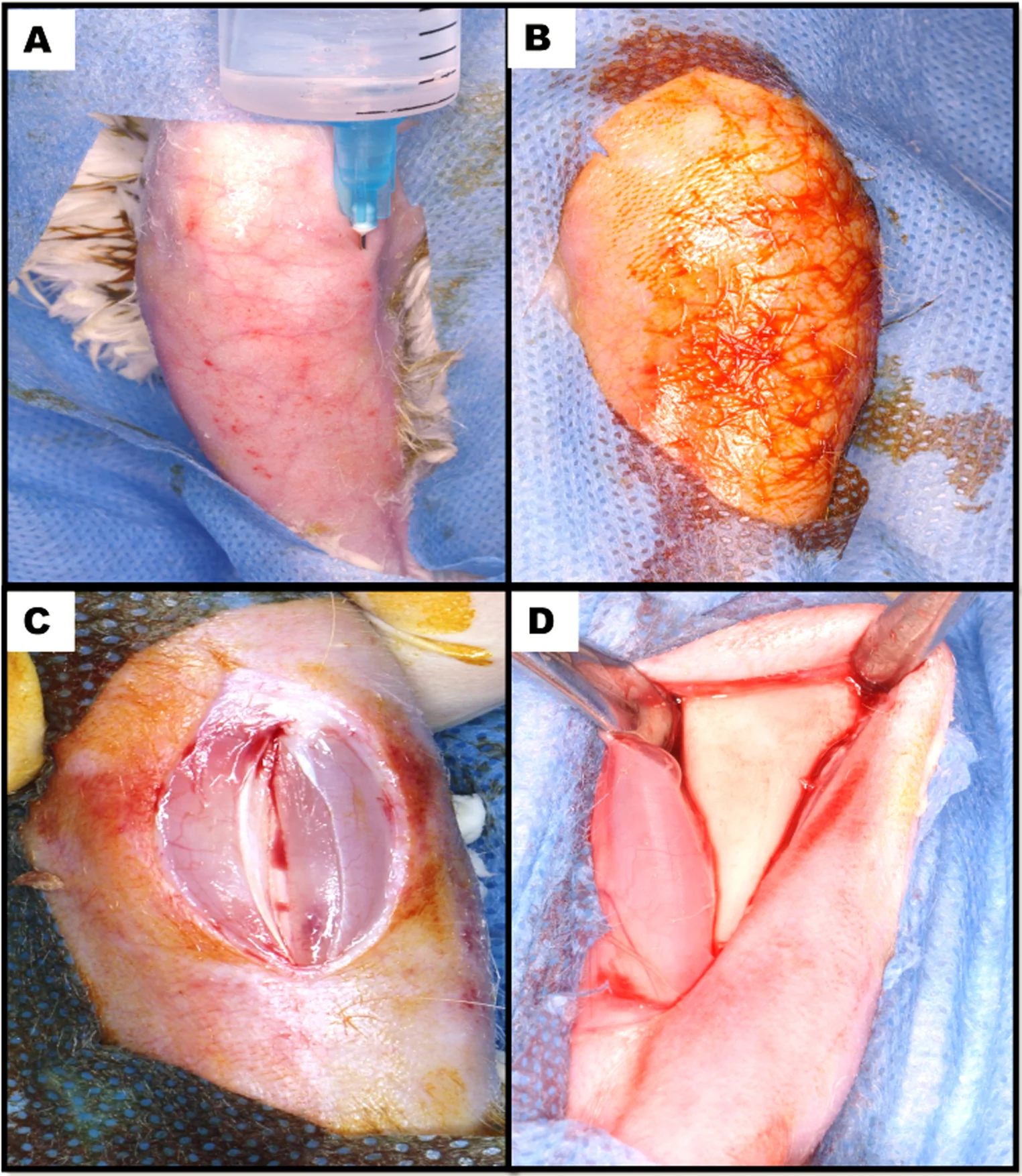
Surgical procedure of tibial bone exposure. (**A**) local anesthesia administration. (**B**) isolation of the surgical field with sterile drapes and disinfection of the field. (**C**) incision through each layer till bone was reached. (**D**) exposure of the tibia after full-thickness flap reflection

All surgical procedures in the current study were performed by a single operator to ensure consistency and minimize inter-operator variability in the surgical technique. General anesthesia was firstly induced by intramuscular administration of (ketamine, 35 mg/kg; Rompun 5 mg/mL) to achieve systemic sedation and analgesia. The surgical areas –skin over both tibiae– were then shaved to facilitate access to the skin and the underlying tissues. For proper analgesia and hemostasis of the surgical theater, local anesthesia (Articaine HCl 68 mg/1.7 ml – Epinephrine 0.017 mg /1.7 ml) was also administered 5 min before the incision. The operative field was subsequently isolated by sterile surgical drapes and disinfected with iodine. The tibial bones were exposed via incision through skin, fascia, muscle, and periosteum layers, followed by their reflection using a muco-periosteal elevator [24] (Fig. 1).

Using the Dual implant system kit and based on the standard recommended parameters (speed 800 rpm and torque 30 N.cm), drilling was started with the point drill, followed by the pilot drill (2.2 mm diameter) and three shaping drills (2.8 mm, 3.4 mm, and 3.8 mm), respectively, creating three different osteotomies. Subsequently, three (4.1*6 mm) titanium implants (grade 23 titanium alloy with Sandblasted, Large-grit, Acid-etched (SLA) surface treatment) were inserted in the tibiae of each rabbit bilaterally [24].

#### CAP treatment procedure

CAP treatment for implants in the CP and CP-M groups was performed immediately before implant insertion using the PiezoBrush^®^ PZ3 (Relyon Plasma GmbH, Regensburg, Germany). The near-field nozzle (module near-field) with a dielectric-barrier cap was selected for implant surface treatment. The device was set at 50 kHz with a power of 8 W. The implants served as the ground electrode. The device was positioned with a working distance of 2 mm between the nozzle and the surface to be treated. The time was set at (countdown, 2 min), and the control signal on the display showed plasma activation. To ensure thorough plasma surface treatment, the implant was attached to the rotary driver and was permitted to spin under the plasma source at a speed of 25 rpm [25].

#### Maca extract application

Following CAP treatment, the implants assigned to the CP-M group were immersed in the prepared Maca nano-suspension for 1 min. The implants were then removed from the nano-suspension and immediately inserted into the prepared osteotomies to minimize any potential alteration of the treated surface before implantation.

#### Implant placement

**Fig. 2:**
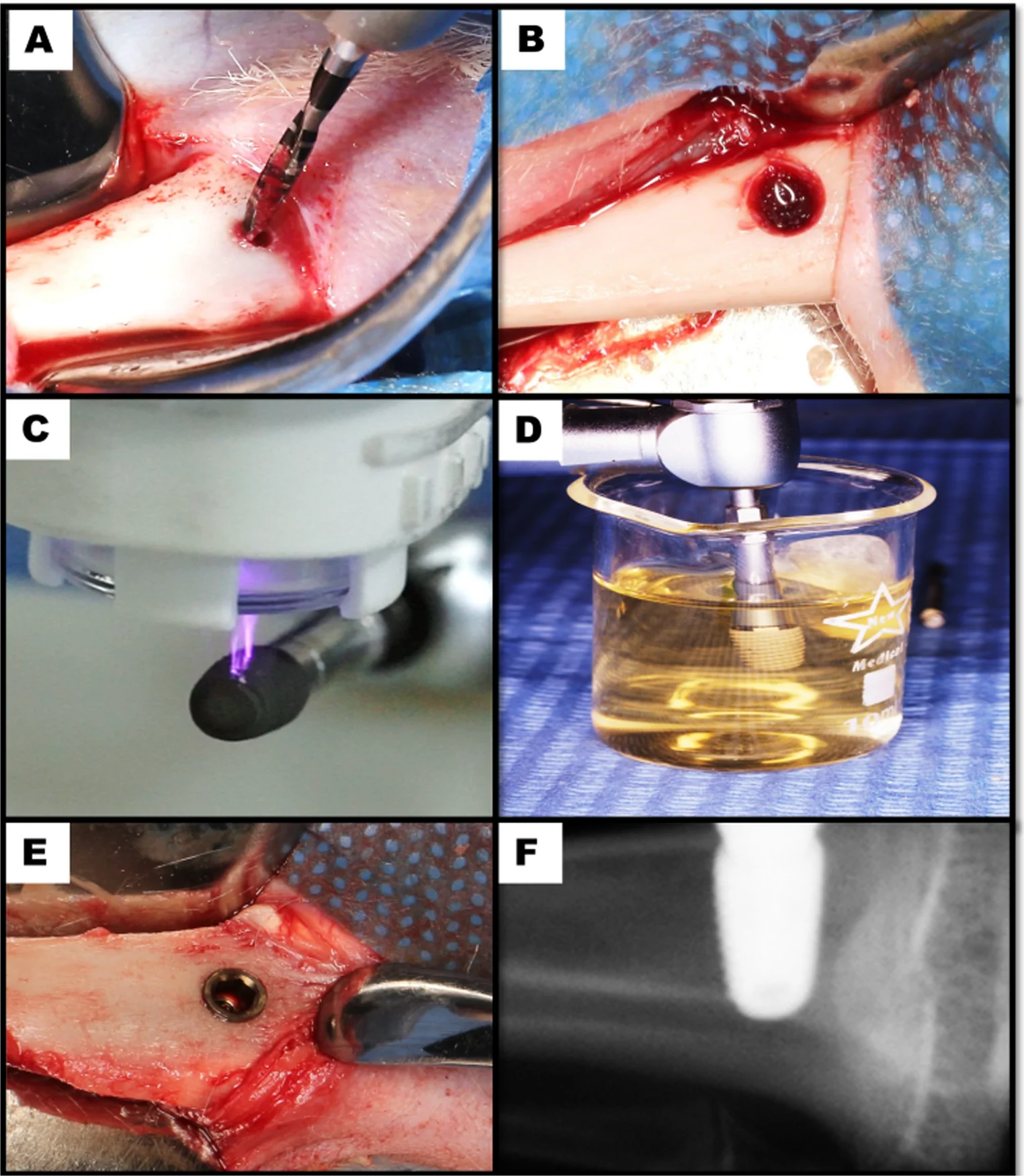
Surgical procedure of implant insertion. (**A**) determination of the implant position with the point drill. (**B**) final osteotomy after shaping drills. (**C**) plasma surface treatment. (**D**) immersion of the implant in the Maca extract for one minute. (**E**) the implant is immediately inserted into the osteotomy after the dual treatment. (**F**) a periapical radiograph shows the position of the implant in the left tibia

In the right tibia, two implants were inserted with an inter-implant distance of 10 mm; the cranial implant represented the CN group, while the caudal implant represented the CP group. In the left tibia, the third implant was inserted, representing the CP-M group. Subsequently, periapical radiographs were obtained for each tibia to verify implant positioning (Fig. 2).

#### Implant primary stability recording

Implant primary stability value was evaluated using the AnyCheck device, which determines implant stability through a non-invasive percussion-based measurement. The device applies a controlled tapping force to the implant/abutment and analyzes its damping response. The resulting damping value (DV) represents the stability of the implant, with higher values indicating greater implant stability. AnyCheck was selected because it allows rapid and non-invasive assessment of implant stability, which was particularly suitable for the present animal model and permitted assessment at both implant placement and four weeks postoperatively without disturbing the healing tissues. According to the manufacturer’s recommendations; a 4 mm long implant’s healing cap was screwed to each implant while the Anycheck device was held at a contact angle of 0°-30° upward while measuring [26]. The obtained values for each implant were recorded as an indicator of the primary stability. Subsequently, the healing cap was removed and replaced by the implant’s cover screw.

#### Wound closure and postoperative care

The surgical site was thoroughly irrigated with saline before closure. The wound was sutured in two layers to optimize the healing. Firstly, the deep muscular layer was stitched with 3 − 0 Vicryl suture, followed by skin closure with lock stitches using the same suture material. Subsequently, the wound was disinfected one more time with iodine and sprayed with antibiotic spray Bivatracin^®^150 ml (Acdima International Trading, Egypt). Finally, postoperative analgesics and antibiotics, as well as wound disinfection, were provided by the veterinary doctor and workers in the animal house in this experimental period of 4 weeks, as shown in Table 1.

**Table 1:** A list of the postoperative medication types, administration dosage, frequency and rout

Agent/substance	Drug	Dosage	Frequency	Route of administration
Antibiotic	Amoxycillin	7–15 mg/kg	Every 24 for 5–7 days	Intramuscularly
Analgesic agent	Paracetamol	1 ml drug/100 ml	3 times /day for the first five days following the surgery	Added to drinking water

#### Implant secondary stability recording

At 4 weeks, all surgical sites were clinically evaluated and showed primary closure with no evidence of wound dehiscence, infection, or other clinically evident postoperative complications. The animals were then sedated, and the surgical sites were reopened to expose the implants. Secondary implant stability was then assessed using the AnyCheck device while the animals were still alive. Following completion of the stability measurements, the animals were sacrificed by a lethal injection of an anesthetic solution consisting of Ketamine and Xylavex (35 mg/kg) intramuscularly [26, 27].

#### Implant removal and removal torque assessment

The implants were removed by reverse rotation using a JDTorque^®^ dynamometric ratchet (JDentalCare, Italy), with a maximum measurable torque of 80 N·cm. The torque required to initiate implant unscrewing was recorded in N·cm when within the measurable range. Removal torque was recorded as a secondary mechanical observation during implant retrieval for histological processing [28]. Measurements exceeding the instrument’s upper limit were recorded as > 80 N·cm. Measurements affected by movement of the surrounding bone during implant removal were considered unreliable and excluded from quantitative analysis.

**Fig. 3:**
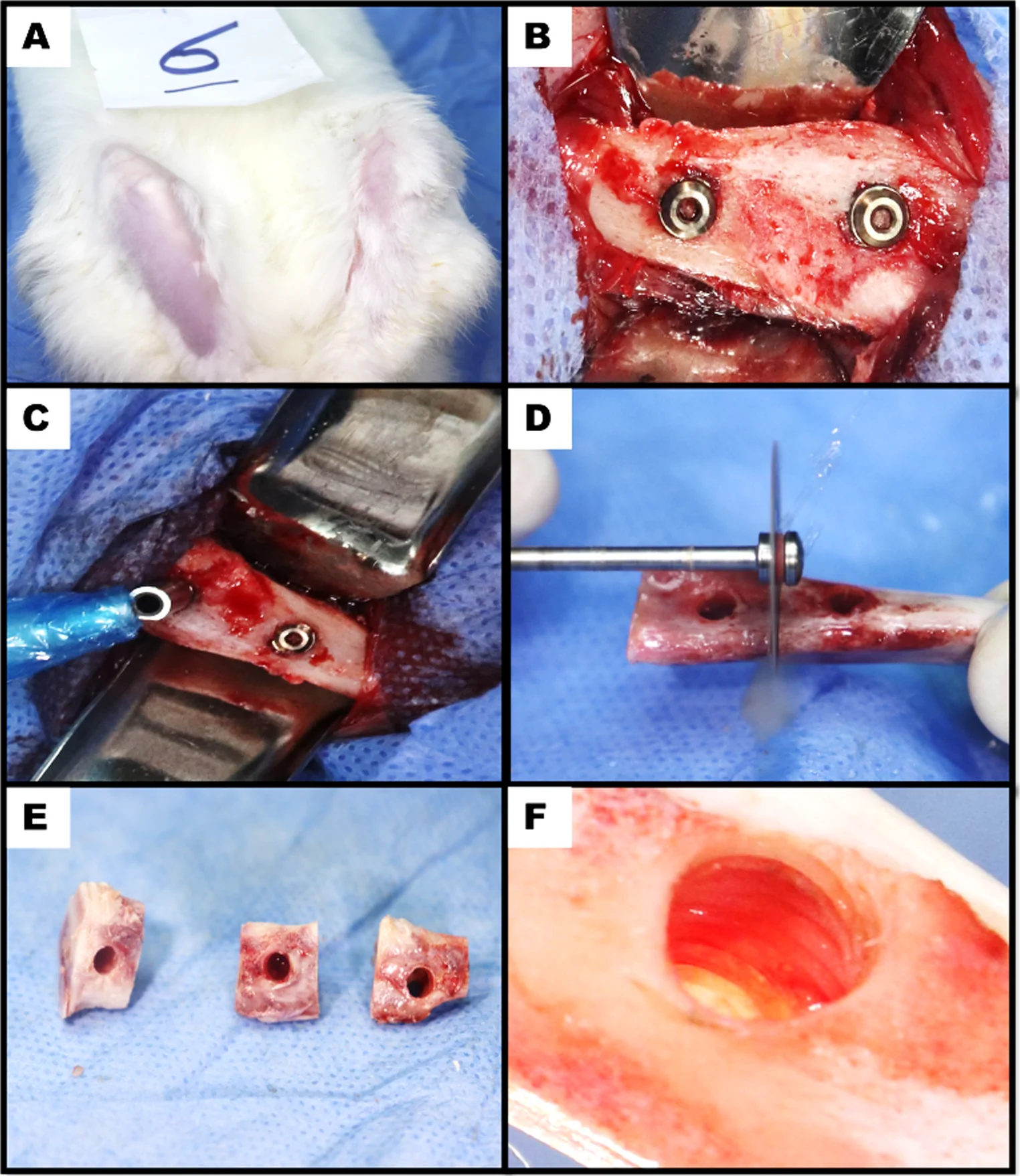
Procedure of secondary stability measurement. (**A**) healing of the surgical sites. (**B**) exposure of the implants. (**C**) obtaining values of secondary stability using the AnyCheck device. (**D**) metal disc was used to prepare bone blocks. (**E**) three bone blocks around each osteotomy after implant removal. (**F**) a magnified photo showing the imprint of the implant’s threads after implant removal

Finally, the tibia was isolated; a block of bone around each implant was sectioned (10 mm in width and 8 mm in length). The bone specimens were sectioned using a metal disc under continuous saline irrigation, with intermittent cutting and brief pauses to prevent excessive heat accumulation and minimize thermal damage to the surrounding bone tissue. Each specimen was then immediately immersed in a jar of 10% formaldehyde for the subsequent specimen processing (Fig. 3).

### Tissue preparation and histomorphometric analysis

Following dissection, the bony specimens were fixed in 10% neutral buffered formalin for 24 h. The samples were then decalcified in EDTA solution and subsequently processed using routine histological procedures. This included dehydration in ascending concentrations of ethyl alcohol (70%, 90%, and 100%). They were then cleared in xylene and embedded in paraffin blocks. Subsequently, the technician assigned the bone specimens from all groups random numerical codes (1–30), thereby blinding the pathologist performing the histological and histomorphometric assessments and the statistician to treatment allocation. For histological examination and histomorphometric analysis using hematoxylin and eosin (H&E) and Masson’s trichrome (MT) staining, 4-µm-thick sections were precisely obtained from the paraffin-embedded blocks using a rotary microtome. A light microscope (Leica Digital Microscope, Leica Microsystems, Wetzlar, Germany) was used for assessment of stained sections for each sample. Image analyzer software (Leica QWin 500 image analysis program, Leica Microsystems, Balgach, Switzerland) was utilized to automatically measure the percentage of any tissue surrounding the implant space. All sections were histologically inspected using (x100) magnification by a single blinded pathologist.

For each specimen, a longitudinal section was obtained from the center of the bone block, parallel to the long axis of the implant bed, so that the entire implant space appeared as a continuous space with all thread serrations visible from the coronal to the apical end. Measurements of three different non-overlapping fields at (x100) in each specimen corresponding to its coronal, middle, and apical thirds, thereby allowing assessment along the entire implant length. The fields were automatically measured by software with a standardized protocol applied to all specimens in all groups. Then the measurements of the 3 fields were averaged to obtain a single value per specimen [29, 30]. The area percentages of newly produced osteoid/osseous bony tissue were measured separately, together with the soft tissue percentage in each sample. Because the implants were removed before decalcification and histological processing, these measurements were considered an assessment of peri-implant bone formation rather than direct bone-to-implant contact (BIC).

### Statistical analysis methods

Data were analyzed using IBM SPSS^®^ (Statistical Packages for Social Sciences, version 25 MB Inc., Chicago, IL). Data was initially explored by Kolmogorov-Smirnov and Shapiro-Wilk tests as normality tests. Since data was normally distributed, it was reported as mean and standard deviation, then the comparison between the three different groups was performed using a one-way ANOVA test, followed by Tukey`s Post Hoc test for multiple comparisons. *P*-value ≤ 0.05 was considered statistically significant.

## Results

### Results of the Maca-NS characterization

The DLS analysis revealed that the Maca-NS exhibited a mean particle size of 246.7 ± 18.23 nm and a PDI of 0.5 ± 0.023. The ZP was found to be -18.7 ± 1.15 mV.

### Stability test

Results of the descriptive statistical analysis for the primary and secondary stability are listed in Table 2. Regarding the primary implant stability, One-way ANOVA for intergroup comparisons revealed no statistically significant difference in the implant stability test (IST) mean values between the study groups. On the other hand, one-way ANOVA revealed a statistically significant difference between the groups regarding the secondary implant stability outcome (*P*-value = 0.015). A Tukey Post Hoc test for pairwise comparison revealed a statistically significant difference between the CP and CN groups only (*P*-value = 0.017). Furthermore, a paired t-test was used for intragroup comparison between the primary and secondary stability, and a statistically significant difference was noted within all groups.

**Table 2:** Showing the results of descriptive analysis and one-way ANOVA test of stability

Outcome	CN group	CP group	CP-M group	*P*-value
Primary stability	60.20 ± 15.50^a^	72.20 ± 7.18^a^	69.80 ± 13.89^a^	0.100
Secondary stability	78.00 ± 11.13^a^	87.70 ± 3.16^b^	85.80 ± 5.20^a&b^	0.015*

*Significance level is equal to or less than 0.05

Means with different superscript letters are significantly different (Tukey’s post hoc test)

### Removal torque assessment

Removal torque was recorded during implant retrieval as a secondary mechanical observation. Measurements were available for nine implants in each experimental group; one implant per group was not included because removal torque was not recorded during its retrieval. The available measurements are summarized in Table 3.

**Table 3:** Representing descriptive analysis of removal torque measurements

Group	*n*	Mean ± SD (*N*·cm)	Median (*N*·cm)	Range
CN	9	48.3 ± 22.9	50	15–80
CP	9	58.3 ± 20.6	60	15–80
CP-M	9	60.6 ± 17.9	60	30–80

### Microscopic examination of the histological sections

**Fig. 4:**
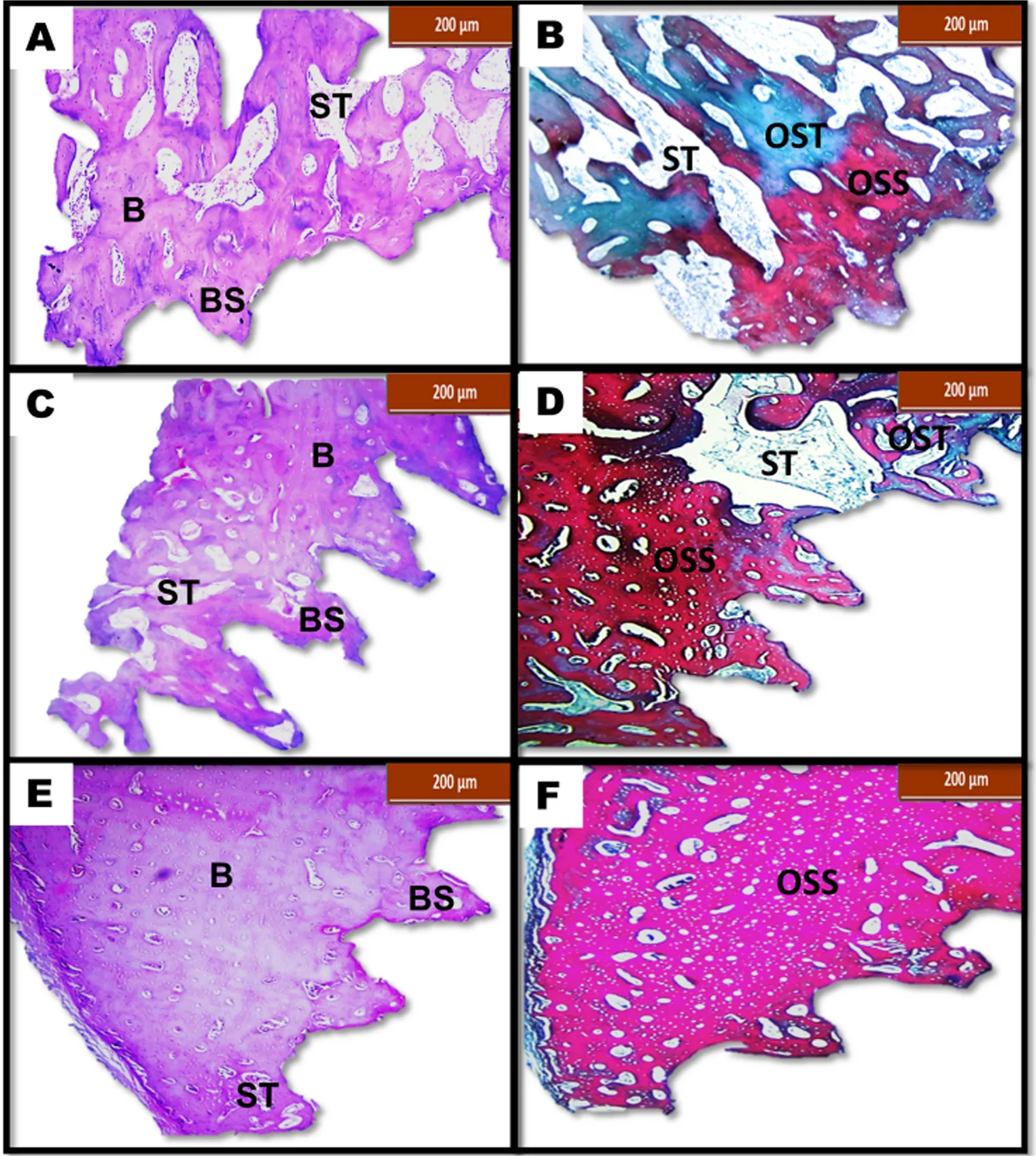
Photomicrographs of histological stained sections for the 3 groups. (**A**) section for CN group showing thin trabeculae of newly formed bone (mainly formed of blue-green osteoid bone) separated by wide marrow spaces filled with fibrovascular connective tissue, and short bone spikes facing implant space are obviously seen in (**B**), sections for CP group displaying thick bone trabeculae (**C**) (mixture of green osteoid and red osseous bone) separated by fibrous tissue. Long and thin bone spikes facing the implant space are clearly detected (**D**), sections for the CP-M group reveal thick areas of newly formed bone trabeculae (**E**) (mainly formed of red osseous bone) separated by small marrow spaces. Thick and dense bone spikes can also be seen facing the implant space (**F**). (A, B,C sections: H&E staining x100, B, D,F sections: MT staining x100) (abbreviations: BS: bone spikes, ST: soft tissue, B: newly formed bone, OST: osteoid bone, OSS: osseous bone)

The inspection of H&E-stained sections of the CN group showed thin, interconnected, newly formed bone trabeculae with short, fragile bone spikes surrounding the implant space (Fig. 4A). Meanwhile, the sections of the CP group revealed thick, newly formed bone trabeculae with long and thin bone spikes surrounding the space of implant serrations. (Fig. 4C). On the other hand, the assessment of the sections of the CP-M group showed thick and dense newly formed bone trabeculae with thick bone spikes clearly seen between the implants’ serration spaces (Fig. 4E).

Regarding the MT-stained sections, examination of the CN group sections revealed interconnected thin bone trabeculae, composed of mixed red osseous and blue –green osteoid tissue, with short red osseous bone spikes surrounding the implant serration space (Fig. 4B). Pointing to the CP group, sections revealed thick bone trabeculae composed of hard red osseous bone and blue-green osteoid bone with thin, long bone spikes surrounding the space of implant serrations (Fig. 4D). On the other hand, the examination of the sections for the CP-M group showed thick and dense bone composed mainly of red osseous bone and minute areas of blue-green osteoid bone with thick osseous spikes between the implant serration spaces (Fig. 4F).

### Histomorphometric analysis

Results of the descriptive statistical analysis for the area percentages of the mineralized and unmineralized newly formed bone, and the soft tissue are listed in Table 4. One-way ANOVA for comparison between the 3 study groups showed a statistically significant difference in the area percentages of newly formed mineralized bone only (*P*-value = 0.018). A Tukey Post hoc test for pairwise comparison revealed a statistically significant difference between CN and CP-M (*P*-value = 0.018).

**Table 4:** Showing the results of descriptive analysis and One-Way ANOVA test of the histomorphometric analysis

Histomorphometric parameter	CN group	CP group	CP-M group	*P*-value
Unmineralized bone%	28.20 ± 12.03^a^	13.38 ± 9.19^a^	16.72 ± 7.49^a^	0.070
Mineralized bone%	28.84 ± 10.39^a^	45.32 ± 9.81^a&b^	50.30 ± 11.27^b^	0.018*
Soft tissue %	10.92 ± 4.09^a^	11.36 ± 6.17^a^	9.76 ± 6.49^a^	0.900

*Significance level is equal to or less than 0.05

Means with different superscript letters are significantly different (Tukey’s post hoc test)

## Discussion

Optimizing early osseointegration remains a significant challenge in implant dentistry, particularly in situations requiring accelerated healing or early loading. CAP has emerged as a promising surface modification strategy because it effectively removes hydrocarbons, increases surface energy, and enhances titanium hydrophilicity, thereby promoting protein adsorption and early osteoblast attachment [5, 31]. Unlike conventional methods such as sandblasting and acid-etching, which require manufacturing-level interventions, CAP can be applied chairside immediately before implant placement, offering a practical and clinically translatable approach [32]. By eliminating organic contaminants, improving wettability, and facilitating osteoblast recruitment and adhesion, CAP has been shown to significantly enhance bone-to-implant contact and secondary stability. These advantages justified its selection as the primary surface treatment in this study. However, plasma activation does not inherently provide sustained osteogenic stimulation [5, 7].

The inclusion of Maca extract as a biological adjunct in this study was based on previous reported evidence of its osteogenic, antioxidant, and anti-inflammatory properties. In vitro study of Liu et al. (2025) has shown that certain Maca constituents can promote osteoblast differentiation and proliferation, by elevating specific proteins (e.g., BMP-2, ALP) [33]. Additionally, another animal study using non-implant conditions has reported Maca’s ability to ameliorate inflammation and improve bone mineral density [11]. Maca was prepared as a nano-suspension to enhance dissolution and bioavailability. The particle size of the prepared suspension confirmed that the Maca was within the nano range, with a mean particle size (246.7 ± 18.23 nm). Such particle size lies within the favorable range reported by several studies for enhanced osteogenic cell attachment and differentiation [34]. Although the precise mechanisms underlying the osteogenic effects of Lepidium meyenii (Maca) remain incompletely understood, several potential mechanisms have been proposed. Maca contains a variety of bioactive constituents, including macamides, macaenes, and glucosinolates, which may contribute to its biological effects [13]. Experimental evidence has suggested that specific Maca-derived compounds may directly influence osteogenic differentiation. For example, N-(3-methoxybenzyl)-(9Z,12Z,15Z)-octadecatrienamide, a macamide isolated from Maca, promoted osteogenic differentiation and bone formation, with increased expression of osteogenic markers including RUNX2, Osterix, and alkaline phosphatase, and was associated with activation of the canonical Wnt/β-catenin signaling pathway [35]. In addition, the antioxidant properties of Maca may provide a favorable environment for bone formation by reducing oxidative stress; a recent systematic review and meta-analysis reported beneficial effects of Maca on several markers of cellular oxidative stress, including increased glutathione, glutathione peroxidase, and superoxide dismutase and decreased malondialdehyde [36]. Thus, the increased mineralized bone area observed in the Maca-treated group in the present study may reflect a broader stimulatory effect on the bone-forming environment rather than an effect restricted to a single stage of bone formation. Nevertheless, because the present study did not investigate specific molecular signaling pathways or osteogenic markers, these mechanisms should be considered possible explanations rather than definitive conclusions. Further molecular and cellular studies are warranted to clarify the pathways through which Maca may enhance bone formation. Based on these findings, this study investigated whether combining CAP with Maca extract as a novel approach could enhance peri-implant bone healing beyond plasma treatment alone.

Adult male New Zealand White rabbits were selected to minimize biological variability and enhance data consistency, as the inclusion of female sex may act as a confounder due to hormonal fluctuations [37]. Only skeletally mature rabbits (> 6 months of age) weighing 2.5–3.0 kg were included to ensure physiological stability and adequate tolerance to the surgical intervention [38]. The tibia was chosen as the implantation site due to its favorable anatomical characteristics, including sufficient cortical bone thickness to permit standardized implant placement and ensure reproducible surgical and histological conditions. Previous literature has confirmed the reliability of the rabbit tibia as an experimental site for osseointegration studies [39, 40].

All implants were commercially pure titanium with an SLA surface, a well-documented modification that enhances bone–implant contact compared with machined surfaces [41]. Implant geometry was standardized (4.1*6 mm) to fit the anatomical limitations of rabbit tibias while still providing sufficient bone–implant contact for biomechanical and histological assessment [42].

Implant stability was assessed using the AnyCheck device as a mechanical indicator of implant stability during the early healing period owing to its reliability and practicality in small animal models with the limited implant size [43]. This provided a complementary assessment to the histological evaluation of peri-implant bone formation. According to the current finding, the primary stability did not differ significantly among groups at the time of implant placement. This may be attributed to the standardized surgical technique, bone density, and implant geometry. Similar observation has been reported in earlier studies conducted by Romero-Serrano et al. (2024) who concluded that implant surface roughness does not affect primary stability but will greatly influence the secondary stability [44].

After four weeks, secondary stability values were significantly higher in the CP group compared to the CN group, and no statistically significant difference between the CP-M and CP groups. This suggests that these interventions can enhance bone healing and remodeling around the implants. These results align with a randomized controlled trial conducted by Makary et al. (2024) that showed that plasma-treated implants had better bone-implant contact; it revealed bone-implant contact in the plasma-treated group of 38.7% versus 22.4% for non-treated surfaces at 4 weeks [8]. Also, in a clinical retrospective study of plasma activation, implants treated with plasma exhibited significantly higher implant stability quotient values at weeks 1, 3, and 4 compared to non-treated ones, thus revealing that plasma treatment positively enhanced clinical implant stability [9].

Histomorphometric analysis provided valuable insight into the quality of the newly formed peri-implant bone by using MT-stained sections and evaluated the biological effects of the interventions. The CP-M group demonstrated the most favorable histological profile, with a significantly higher mean value of dense mineralized bone surrounding the former implant serration spaces. However, because the implants were removed before decalcification and paraffin processing, the present histological method did not permit direct quantitative assessment of BIC. Therefore, the observed histological differences should be interpreted as differences in peri-implant bone formation rather than direct evidence of the percentage of bone in contact with the original implant surface. This may be attributed to the fact that CAP enhances early protein adsorption and cellular attachment through increased wettability, while Maca provides bioactive compounds that promote osteoblast differentiation and modulate inflammatory responses during the critical early healing phase [5]. The osteogenic and anti-inflammatory effects of Maca observed in the present study are consistent with the findings of a recent study conducted by Abdoun et al. (2026) [14]. On the other hand, the optimized findings of using nano-formulated coating were in accordance with those reported by Rehner et al. (2025) [45].

The presented findings are in similar to the results reported by Ashtiani et al. (2022), who illustrated that Grape seeds extract (known for its potent anti-oxidant and anti-inflammatory effects) coating appeared as a “biological bridge,” that potently increased the percentage of bone directly anchored to the titanium implant surface compared to the uncoated implants group [10]. On the opposite side to this current finding, a previous study illustrated that implants coated with BMP-2 or VEGF (known for their capability of enhancing osteoblastic differentiation and proliferation) have no significant effect on implant -bone contact histologically compared to untreated implant groups [46]. These contradictory results observed with Maca extract suggest that some biological coatings, such as collagen I and BMP-2 coating, are actually underpowered when applied to the implant surface [47]. Given that the beginning of the bone remodeling process was initiated after implant placement, when osteoclasts begin the process of osteoclastogenesis of old bone (that ensures primary stability), followed by phase of the formation of new bone around the implant surface, thus prolonged time (> 8 weeks) before testing secondary stability, is the most suggested the key factor in obtaining better significant values of secondary stability in CP-M group, especially that it showed better significant values regarding newly formed mineralized bone [48]. Also, an alternative study highlighted that there were no clinically noteworthy differences between titanium implants coated with recombinant human growth/differentiation factor and the uncoated implants, although it showed significantly better histological results regarding implant bone contact percentage [49].

Thus, combining physical surface activation with biologically active adjuncts may represent a rational strategy to optimize early implant integration and long-term success of the osteointegration process, shorten healing periods, and reduce the risk of early implant failure [50]. As a pilot in-vivo investigation of a novel combination of CAP and Maca nanosuspension, the present study provides preliminary evidence regarding its potential effect on peri-implant bone formation. These findings should therefore be interpreted as exploratory and hypothesis-generating, rather than as definitive evidence of efficacy. The results may provide a basis for future studies with larger sample sizes, longer follow-up periods, and more comprehensive biological and surface characterization.

### Limitations and future perspectives

Finally, several limitations of this study should be acknowledged. As a pilot in-vivo investigation of a novel intervention, the relatively small sample size limits the statistical power and generalizability of the findings, which should therefore be considered preliminary and used to inform the design of future adequately powered studies. Although the rabbit tibia model is suitable for investigating early osseointegration, it does not fully replicate the biomechanical environment of the human jaw, and the four-week follow-up was limited to early healing outcomes. The fixed allocation of implant treatments to specific tibial sides may also introduce anatomical side-related confounding, despite being intentionally adopted to minimize potential local cross-effects of the Maca treatment. Furthermore, because the implants were removed before decalcification, histological assessment focused on peri-implant bone formation rather than direct BIC measurement. Future studies should therefore incorporate longer follow-up periods, larger sample sizes, direct BIC assessment using undecalcified sections, and quantitative characterization of the amount, distribution, and retention of the Maca nanosuspension on the implant surface following application and insertion.

## Conclusion

The outcomes of the current study highlight the value of CAP as a simple, chairside surface modification of implants. The incorporation of a nano-formulated natural extract such as Maca may further enhance the biological response at the implant–bone interface. Such a combined surface treatment strategy may offer a promising novel, cost-effective approach to improve implant bioactivity and support early osseointegration, highlighting the potential of integrating physical surface modification with natural biomolecular agents.

## Data Availability

The data supporting the findings of this study are available from the authors upon reasonable request.
